# Specific Detection of SARS-CoV-2 Variants B.1.1.7 (Alpha) and B.1.617.2 (Delta) Using a One-Step Quantitative PCR Assay

**DOI:** 10.1128/spectrum.02176-21

**Published:** 2022-03-14

**Authors:** Oran Erster, Ella Mendelson, Areej Kabat, Virginia Levy, Batya Mannasse, Hadar Assraf, Roberto Azar, Yaniv Ali, Efrat Bucris, Dana Bar-Ilan, Orna Mor, Michal Elul, Michal Mandelboim, Danit Sofer, Shay Fleishon, Neta S. Zuckerman, Itay Bar-Or

**Affiliations:** a Central Virology Laboratory, Public Health Services, Ministry of Health, Chaim Sheba Medical Center, Ramat Gan, Israel; b School of Public Health, Sackler Faculty of Medicine, Tel-Aviv University, Tel-Aviv, Israel; Fundacio irsiCaixa

**Keywords:** SARS-COV-2, RT-qPCR, Alpha (B.1.1.7), Delta (B.1.617.2), classification, diagnostic assay, SC-2 surveillance

## Abstract

In this report, we describe the development of a reverse transcription-quantitative PCR (RT-qPCR) assay, termed Alpha-Delta assay, which can detect all severe acute respiratory syndrome coronavirus 2 (SC-2) variants and distinguish between the Alpha (B.1.1.7) and Delta (B.1.617.2) variants. The Alpha- and Delta-specific reactions in the assay target mutations that are strongly linked to the target variant. The Alpha reaction targets the D3L substitution in the N gene, and the Delta reaction targets the spike gene 156 to 158 mutations. Additionally, we describe a second Delta-specific assay that we use as a confirmatory test for the Alpha-Delta assay that targets the 119 to 120 deletion in the Orf8 gene. Both reactions have similar sensitivities of 15 to 25 copies per reaction, similar to the sensitivity of commercial SC-2 detection tests. The Alpha-Delta assay and the Orf8_119del_ assay were successfully used to classify clinical samples that were subsequently analyzed by whole-genome sequencing. Lastly, the capability of the Alpha-Delta assay and Orf8_119del_ assay to identify correctly the presence of Delta RNA in wastewater samples was demonstrated. This study provides a rapid, sensitive, and cost-effective tool for detecting and classifying two worldwide dominant SC-2 variants. It also highlights the importance of a timely diagnostic response to the emergence of new SC-2 variants with significant consequences on global health.

**IMPORTANCE** The new assays described herein enable rapid, straightforward, and cost-effective detection of severe acute respiratory syndrome coronavirus 2 (SC-2) with immediate classification of the examined sample as Alpha, Delta, non-Alpha, or non-Delta variant. This is highly important for two main reasons: (i) it provides the scientific and medical community with a novel diagnostic tool to rapidly detect and classify any SC-2 sample of interest as Alpha, Delta, or none and can be applied to both clinical and environmental samples, and (ii) it demonstrates how to respond to the emergence of new variants of concern by developing a variant-specific assay. Such assays should improve our preparedness and adjust the diagnostic capacity to serve clinical, epidemiological, and research needs.

## INTRODUCTION

The emergence of new severe acute respiratory syndrome coronavirus 2 (SC-2) variants has been a major concern due to their potential to increase morbidity and mortality, thereby causing further difficulty in fighting and containing the worldwide coronavirus disease 2019 (COVID-19) pandemic (1–3). Since the end of 2020, more than 20 SC-2 variants with distinct genomic signatures have been identified worldwide, and some of these variants divided into additional subclades (https://covariants.org/ and https://cov-lineages.org/lineage_list.html). Some of them were characterized by markedly increased infectivity, which led to recurring outbreaks and a “take-over” effect of specific variants, such as B.1.1.7 (Alpha), in a matter of 4 to 5 weeks (1). Other variants, such as B.1.351 (Beta) and P1 (Gamma), spread rapidly and showed a significant potential to become dominant (4, 5) but were eventually pushed aside by others. Variant B.1.617, initially identified in India in late 2020, spread rapidly, and one of its sublineages (B.1.617-2) became a globally dominant variant within a few months. The transmission pattern of Alpha and Delta variants suggests that they both spread from their country of origin, rapidly reaching North and South America, Europe, Africa, and Australia (4, 5). In Israel, two large morbidity waves were associated with the introduction of new variants. The first wave was the Alpha variant high-morbidity wave, which started in mid-December 2020 and declined by mid-March 2021 following the successful nation-wide immunization campaign with two doses of the BNT162-b2 vaccine (6). The current high-morbidity wave dominated by the Delta variant followed, which started in July 2021 in parallel to vaccine waning immunity and continues (7). Immunological studies demonstrated that some of the emerging variants have the potential to evade, at least partially, the neutralizing effect of both convalescent patients and vaccinated individuals (2, 3, 7–9). The spreading of such variants may therefore compromise the effectiveness of global vaccination efforts aimed to contain the COVID-19 pandemic.

A fundamental pillar of the campaign against COVID-19 is the ability to detect the presence and identity of SC-2 RNA in both infected individuals and environmental samples. This pressing need led to an unprecedented number of commercial detection kits based on molecular and serological principles, with various degrees of sensitivity, throughput, reliability, and turnaround time (see references 10–12 and references therein). Due to their epidemiological and medical importance, the rapid identification of circulating variants becomes a central tool in the fight against the pandemic.

In addition to the diagnosis of clinical samples, the monitoring of SC-2 RNA in environmental samples, mostly, but not exclusively, wastewater, proved as an important tool (6, 13, 14). Detection of SC-2 RNA in such samples is exceptionally challenging due to their very low abundance and the presence of PCR inhibitors, which render this approach even more difficult. Several reports describe the successful identification of SC-2 RNA in environmental samples, and some describe the classification of the examined samples using sequencing (6, 15, 16). Classification of samples using sequencing requires sufficient quality and quantity, which are often not available with environmental samples. Moreover, this approach is currently much more expensive than PCR-based assays and cannot be scaled-up readily to facilitate rapid screening of a large number of samples efficiently.

In order to address the need for rapid classification of SC-2-positive samples, several manufacturers released sets of molecular tests that specifically detect key mutations that are associated with increased transmissibility or vaccine resistance (Seegene, Thermo Fisher, and Kogene Biotech). However, most of these mutations are common to multiple variants and can therefore not be used to assign a sample of interest to a certain lineage with a high degree of confidence using a single test. We recently developed quantitative PCR (qPCR)-based molecular tests that target mutations that are strongly associated with variants Alpha and Beta, thereby enabling rapid classification of such samples with high confidence. We demonstrated that the selected target mutations successfully reflect the lineage of the examined sample, as confirmed by Sanger and whole-genome sequencing (17). In this report, we describe the development and utilization of two qPCR tests that target mutations that are, thus far, unique to variant B.1.617 (Delta lineage). We combined one of these reactions with the N_D3L_ reaction that detects the Alpha variant and the inclusive E-sarbeco reaction (18) we described previously to a new selective multiplex assay. This assay can determine if the examined samples are of lineage Alpha, Delta, or neither. We show that the new multiplex assay, which we term “Alpha-Delta assay,” is rapid, sensitive, and reliable, as confirmed by sequencing. Finally, we demonstrate the capability of the new assay to detect and classify SC-2 RNA extracted from environmental samples.

## RESULTS

### Design of the S_157del_ and Orf8_119del_ reactions.

**FIG 1:**
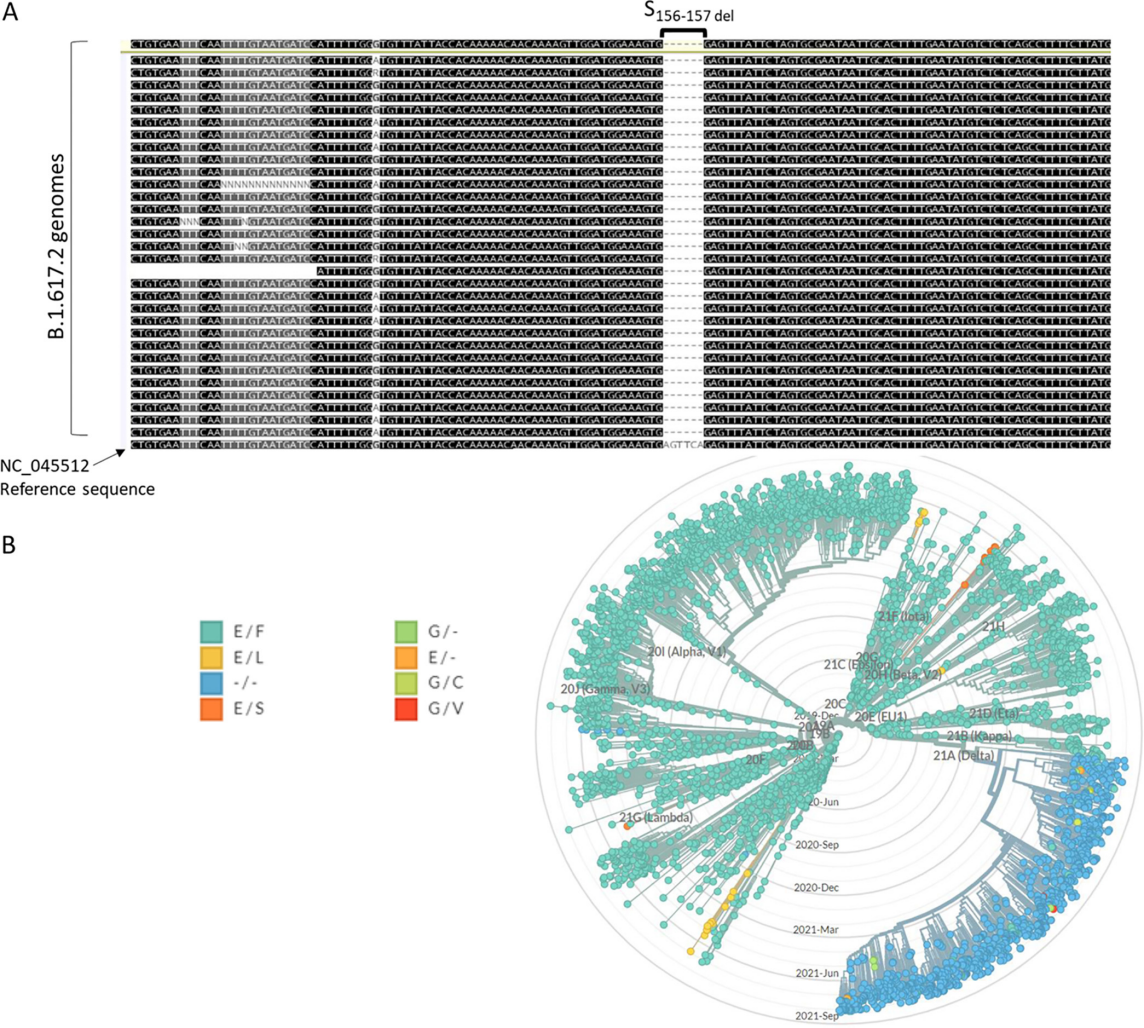
Identification of the B.1.617-specific spike gene deletion. (A) Alignment of 32 genomic sequences of lineage B.1.617 samples with reference sequence NC_045512 showed a deletion in nucleotide positions 22045 to 22050, corresponding to amino acid positions 156 to 157 in the protein sequence. The 156-to-157 deletion gap is marked with brackets. (B) Global analysis of SC-2 genomes available in the NextStrain database (https://nextstrain.org/sars-cov-2/) showing that this 6-bp deletion is present only in the Delta lineage (highlighted in purple).

Alignment of genomes identified as the Delta lineage from the Global Initiative on Sharing All Influenza Data (GISAID) database (https://www.gisaid.org/) with SC-2 reference sequence NC_045512 showed that all Delta sequences contained a substitution and a deletion in nucleotide positions 22045 to 22050, which translates into amino acid E-to-G substitution in position 156 and deletions in positions 157 to 158 in the spike protein (Fig. 1A). Accordingly, a specific reaction was designed to detect that deletion, denoted S_157del_ reaction hereafter, by using a probe that can only bind to the mutated sequence (21987 probe) (Fig. 1B). In order to establish that the selected mutations were indeed unique to the Delta lineage, global analysis of SC-2 genomes was performed using the NextStrain website (https://nextstrain.org/) using the deletion in positions 22045 to 22050 as a search term. The resulting dendrogram showed that the double deletion was indeed unique to the Delta lineage and was absent from other known lineages (Fig. S1A in the supplemental material).

**FIG 2:**
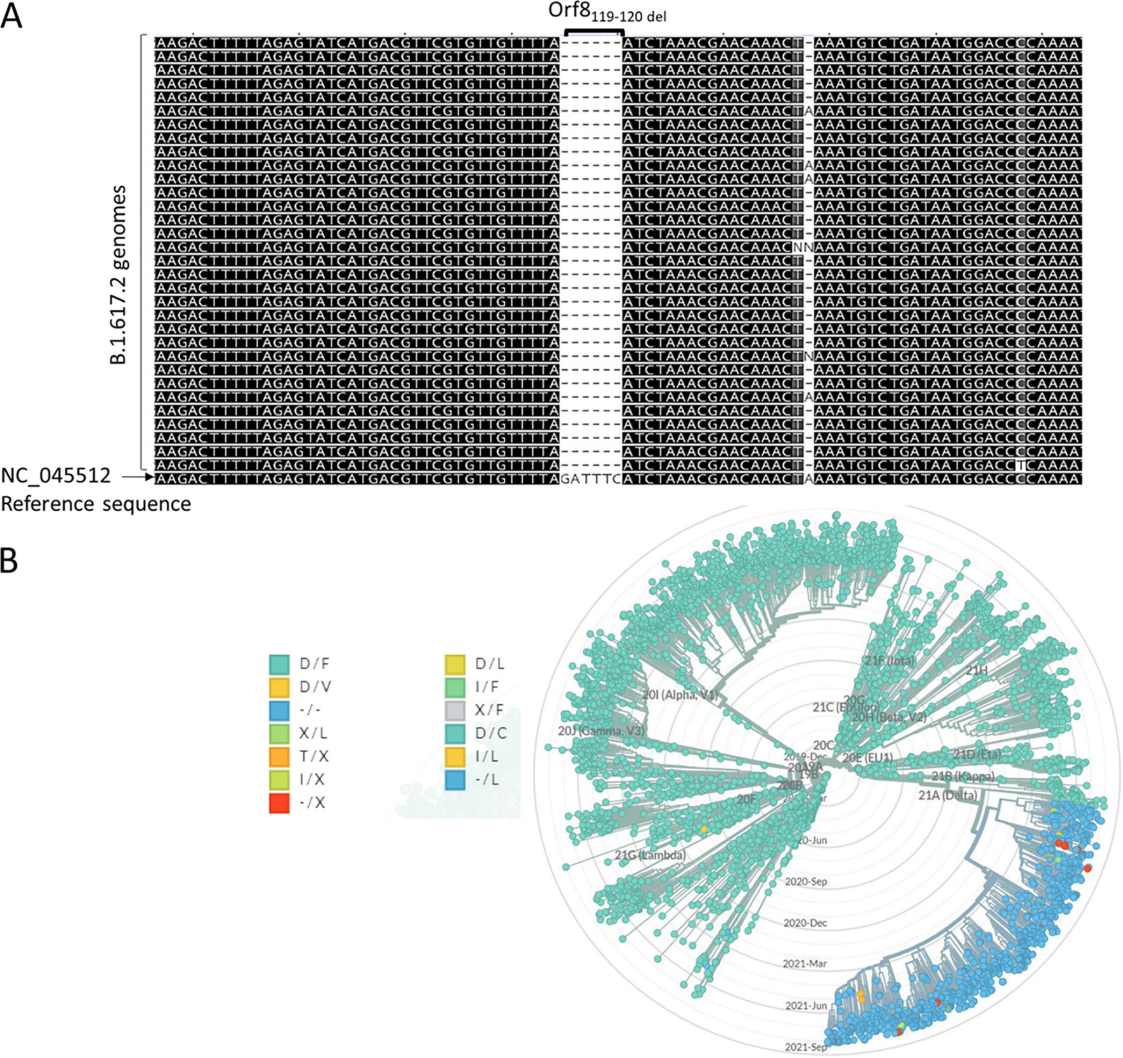
Identification of the B.1.617-specific Orf8 gene deletion. (A) Alignment of 32 genomic sequences of lineage B.1.617 samples with reference sequence NC_045512 showed a deletion in nucleotide positions 28248 to 28253, corresponding to amino acid positions 119 to 120 at the C-terminal end of the protein sequence. The 119-to-120 deletion gap is marked with brackets. (B) Global analysis of SC-2 genomes available in the NextStrain database (https://nextstrain.org/sars-cov-2/) showing that this 6-bp deletion is present only in the Delta lineage (highlighted in purple).

The Delta genome alignment showed a second unique deletion mapped to nucleotide positions 28248 to 28253 in reference sequence NC_045512 at the C-terminal end of Orf8, spanning amino acids 119 to 120 of the protein (Fig. 2A). Global genome analysis performed as described for the 22045-to-22050 deletion showed that the deletion at positions 28248 to 28253 was also unique for the Delta lineage (Fig. 2B). A specific probe was designed to identify the deletion-containing sequence (28199). Secondary structure simulation suggested that the original probe sequence is likely to generate a strong stem-loop structure, thereby significantly reducing the reaction efficiency. In order to avoid this undesired outcome, a single G-to-T substitution was incorporated at position 10 (Fig. S1). Two primers were then designed to amplify the deletion region and complete the reverse transcription-quantitative PCR (RT-qPCR) assay. The sequences of the Orf8_119-120del_ primers and probe are detailed in Table 1. Sequencing of the C-terminal domain (CTD) of the Orf8 gene from the same four samples classified by the S_157del_ assay as Delta confirmed the presence of the Orf8 119 to 120 deletion, which is the target of the Orf8_119del_ reaction (Fig. 2B). Additional confirmation for the accuracy of the two reactions was performed by sequencing four samples that were identified as Delta suspected. Each sample was sequenced in the S_157del_ region and the Orf8_119del_ region. As shown in Fig. S2, all samples contained both deletions.

**TABLE 1:** Details of the primers and probes used for the S_157del_ and Orf8_119del_ assays

Name	Sequence 5′→3′ and modifications^*a*^	Position in sequence NC_045512
S_157del_ reaction		
22003 Fwd	GTGTTTATTACCACAAAAACAACAA	22,003
21987 probe	TexasRed-GATGGACAGTGGAGTTTATTCTAGTG-BHQ2	22,040
22075 Rev	GGCTGAGAGACATATTCAAAAGTGC	22,075 on reverse strand
T7 21494 Fwd	**TAATACGACTCACTATAGG**GGACTTATAATTAGAGAAAACAACAG ^*b*^	21,494
Cov19 22475	GTTTCTGAGAGAGGGTCAAGTGCAC	22,459 on reverse strand
		
Orf_8_ cloning and detection primers		
28225 Fwd	GAAGACTTTTTAGAGTATCATGAC	28,209
28232 Rev	TTTTGGGGTCCATTATCAGAC	28,297 on reverse strand
28199 probe	HEX-CGTGTTGTTGTAATCTAAACGAACAAAC-BHQ1	28,242
pT7 27945 Fwd	**TAATACGACTCACTATAGGG**CCATATGTAGTTGATGACCCGTGTC ^*b*^	27,981
28525 Rev	CCATCTTGGACTGAGATCTTTCATTTTAC	28,598 on reverse strand

E-sarbeco reaction		
E_Sarbeco_F1b	GTTAATAGCGTACTTCTTTTTCTTGC	26,284
E_Sarbeco_R2	ATATTGCAGCAGTACGCACACA	26,382 on reverse strand
E_Sarbeco_P1	6-FAM-ACACTAGCCATCCTTACTGCGCTTCG-BHQ1	26,332


N-D3L reaction		
28257D3L Fwd	TAAACGAACAAACTAAATGTCTCTA	28,257
CoV19_N1-R	TCTGGTTACTGCCAGTTGAATCTG	28,359 on reverse strand
CoV19_N1-P	HEX-ACCCCGCATTACGTTTGGTGGACC-BHQ1	28,309
**RNAse P**	**Sequence 5′→3′ and modifications**	**Position in sequence NM_001104546**
RNase P-Fwd	AGATTTGGACCTGCGAGCG	28
RNase P-Rev	GAGCGGCTGTCTCCACAAGT	49
RNase P-P	Cy5-TTCTGACCTGAAGGCTCTGCGCG-BHQ-2	93

a BHQ, black hole quencher.

b Minimal T7 promoter sequence is in bolded letters.

### Development and evaluation of the Alpha-Delta assay.

**FIG 3:**
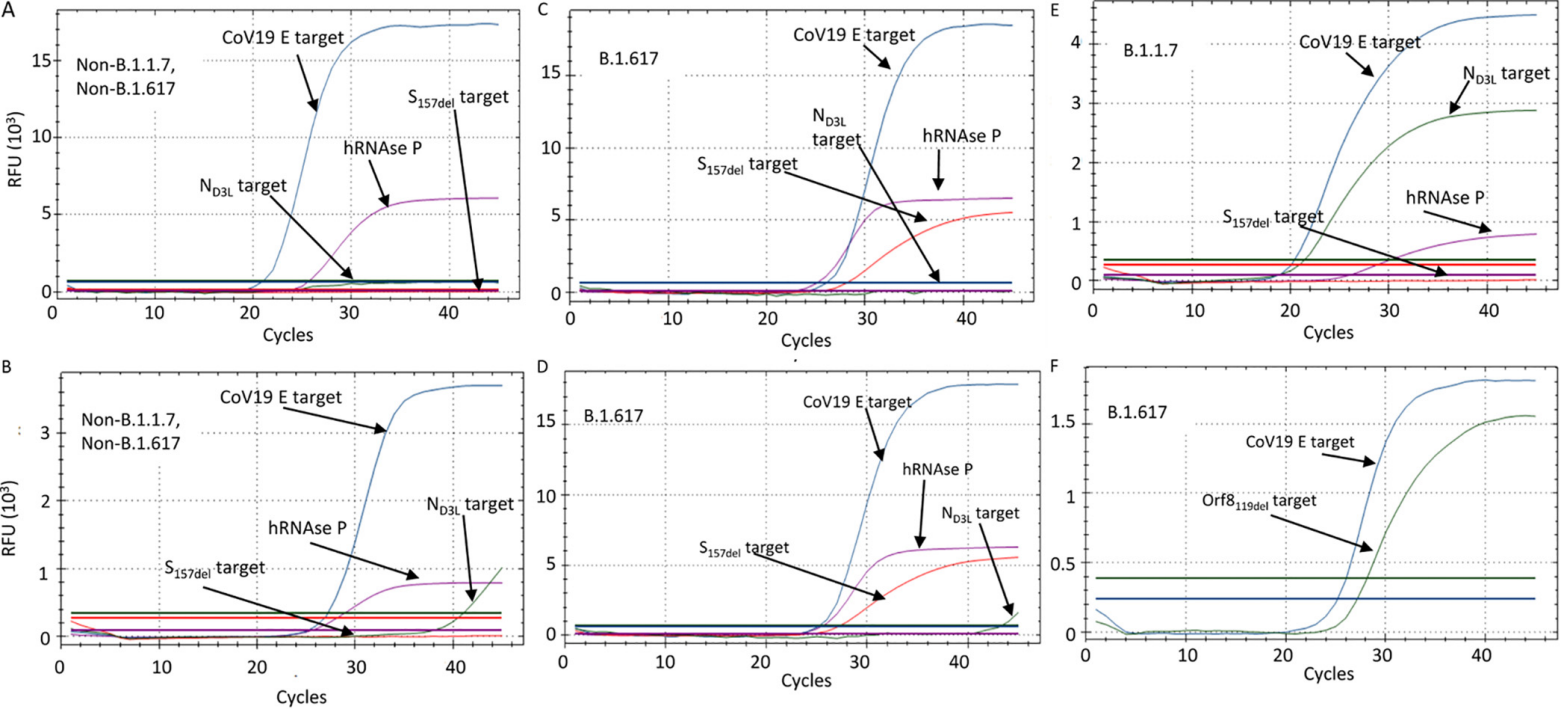
Amplification curves of the Alpha-Delta and Orf8 assays. (A) Non-B.1.1.7, non-B.1.617 sample with no background signal. (B) Non-B.1.1.7, non-B.1.617 sample with N_D3L_ reaction background. (C) B.1.617 sample with no background signal. (D) B.1.617 sample with N_D3L_ reaction background. (E) B.1.617 sample reaction. (F) B.1.617 E-sarbeco + Orf8_199del_ reaction.

In order to facilitate detection of SC-2 RNA regardless of the lineage and identify the Alpha or Delta lineages in a single test, the three SC-2-targeting reactions (i.e., E-sarbeco, N_D3L_, and S_157del_) were combined in one multiplex assay termed “Alpha-Delta assay.” A control reaction detecting the human RNAse P gene was also included in the multiplex assay for an endogenous control, as was performed previously (17). Analytical sensitivity of the Alpha-Delta assay was evaluated using serial dilutions of mixed RNA targets of the four reactions combined in the assay. Each dilution was examined as follows: dilutions down to 5 × 10^3^ copies/reaction were tested in ten replicates. Dilutions of 5 × 10^2^ to 5 × 10^0^ copies/reaction were run in 20 repeats (Table S2). In order to ensure that the calculated number of the *in vitro* transcribed target molecules (IVT standards) provides a reliable estimation of the actual viral genome copies, serial dilutions of commercial SC-2 RNA were tested together with the IVT standards in the E-sarbeco reaction. The combined calibration curve confirmed that the results obtained with the IVT standards are consistent with those obtained with the commercial SC-2 RNA standard (red circled data points, Fig. S3A). The limit of detection (LOD) for the SC-2 targets was determined to be 12 copies/reaction for the E-sarbeco reaction, 50 copies for the N_D3L_ reaction, and less than 10 copies for the S_157del_ reaction. The LOD for the human RNAse P reaction was 50 copies/reaction (Fig. S3). The Orf8_119del_ reaction was developed as a confirmatory assay for inconclusive Delta-suspected samples. This reaction was used in combination with the E-sarbeco reaction that served as an inclusive SC-2 control reaction to ensure the presence of the SC-2 RNA. The analytical LOD of that reaction was determined to be 24 copies/reaction (Fig. S3). The specificity of the Delta-specific reactions was tested using RNA extractions from lineages 19A/19B (Wuhan lineage), Alpha, Beta, and Gamma. As detailed in Table S3, all lineages were negative for both the S_157del_ and Orf8_119del_ reactions. Analytical sensitivity of the multiplex reaction in wastewater was evaluated as described previously (19), and the minimum detection values were close to those obtained with standard sampling material (19, Erster and Bar-Or, unpublished data). Analysis of the amplification curves of the different Delta assay variations showed that in some samples, mostly with a high RNA concentration (quantification cycle [*C_q_*] ≤ 22), a weak signal in the 6-carboxy-2,3,3,5,7,7-hexachlorofluorescein (HEX) channel was observed, presumably resulting from low-affinity N_D3L_ reaction (Fig. 3D). However, in these cases, the *C_q_* values of the E-sarbeco reaction and the S_157del_ reaction (in Delta samples) were always at least 10 cycles earlier than those of the N_D3L_ reaction, thereby clearly indicating that the sample was not of the Alpha lineage. Both the S_157del_ and the Orf8_119del_ reactions were very specific and did not give a positive signal in non-Delta samples (Fig. 3). In order to exclude a possible mutual effect of the four reactions combined together, serial dilutions of Delta RNA from cultured cells were tested, showing similar sensitivity of both the E-sarbeco and S_157del_ reactions, thus confirming the maintained sensitivity of the Alpha-Delta multiplex (Fig. S3).

### Examination of clinical samples using the Alpha-Delta assay followed by whole-genome sequencing (WGS) analysis.

Between January and May 2021, the prevalent lineage in Israel was Alpha. The Delta lineage began spreading in the country during mid-June 2021. The Alpha-Delta assay was therefore used to classify clinical samples whose lineage attribution was unknown in order to enable rapid epidemiological investigations and to aid public health decision-making regarding WGS prioritization. This assay was then used as a routine test at the Israel Central Virology Laboratory. From June to August 2021, over 1,400 clinical samples were classified as Delta, Alpha, or neither using this assay. This classification was subsequently confirmed by Illumina WGS analysis. The details of 62 such samples selected randomly are listed in Tables 2 and 3. All samples classified by the Alpha-Delta qPCR assay as suspected Delta were either identified as such by the WGS analysis or classified as “no lineage” (Table 2). Those who were designated “no lineage” were mostly with *C_q_* values higher than 32 and were all with 70% or less sequencing coverage (Table 3). These samples, which were classified as “Delta-suspected” by the Alpha-Delta assay, were positive for the Orf8 119 to 120 deletion, but the S_157del_ region was not fully sequenced. Comparison of the PCR results with the Illumina WGS analysis showed that of the 62 samples randomly selected, 19 were not classified by the WGS analysis but were positive for the Delta-specific deletions (the S_157del_, Orf8_119del_, or both) (Table 2). Examination of the *C_q_* values obtained for the WGS nonclassified samples showed that two samples had values of 27 and 29 (14,957 and 14,968, respectively), and all others were higher than 30 (Table 2). The sequence coverage of the unclassified samples ranged between 20.9% and 70% (Table 3). These data suggest that the positive identification of both the Orf8_119del_ and the S_157del_ mutations by the qPCR assay enable a more definite classification of these samples, even in the absence of a complete sequencing coverage. The assay also detected a small number of Alpha lineage samples (two in the samples presented in Tables 2 and 3), where the N_D3L_ mutation was detected by both the qPCR assay and the WGS analysis (Tables 2 and 3).

**TABLE 2:** *C_q_* values and classification of the Alpha-Delta assay compared with the Pangolin classification (https://cov-lineages.org/resources/pangolin.html) as determined by the WGS analysis of 62 representative samples^*a*^

Sample	CoV19 E	N_D3L_	S_157del_	ORF8_119del_	Pangolin clade	RT-PCR classification
14918	25		27	26	B.1.617.2	Suspected B.617.2
14920	36		38	40	None	Suspected B.617.2
14921	26		28	26	B.1.617.2	Suspected B.617.2
14922	23		25	23	B.1.617.2	Suspected B.617.2
14923	17		19	18	B.1.617.2	Suspected B.617.2
14924	27		30	27	B.1.617.2	Suspected B.617.2
14925	36		37	34	None	Suspected B.617.2
14926	22		24	22	B.1.617.2	Suspected B.617.2
14928	37		NA	39	None	Suspected B.617.2
14929	20		22	20	B.1.617.2	Suspected B.617.2
14931	31		33	30	B.1.617.2	Suspected B.617.2
14932	34		40	33	None	Suspected B.617.2
14933	21		23	21	B.1.617.2	Suspected B.617.2
14934	29		31	29	None	Suspected B.617.2
14936	35		40	33	None	Suspected B.617.2
14937	21		23	21	B.1.617.2	Suspected B.617.2
14938	34		42	34	None	Suspected B.617.2
14939	33		35	32	None	Suspected B.617.2
14940	28		30	28	B.1.617.2	Suspected B.617.2
14941	24.68	25.51	NA	NA	B.1.1.7	Suspected B.1.1.7
14945	29		31	30	None	Suspected B.617.2
14946	25		28	26	B.1.617.2	Suspected B.617.2
14947	25		27	26	B.1.404	Suspected B.617.2
14948	33		35	33	None	Suspected B.617.2
14949	22		24	23	B.1.617.2	Suspected B.617.2
14950	27		29	28	B.1.617.2	Suspected B.617.2
14951	36		37	35	None	Suspected B.617.2
14952	24		26	25	B.1.617.2	Suspected B.617.2
14953	22		24	22	B.1.617.2	Suspected B.617.2
14954	25		28	21	B.1.617.2	Suspected B.617.2
14955	25		27	26	B.1.617.2	Suspected B.617.2
14957	25		27	26	None	Suspected B.617.2
14958	22		23	22	B.1.617.2	Suspected B.617.2
14960	27		29	27	B.1.617.2	Suspected B.617.2
14963	32		34	32	None	Suspected B.617.2
14965	23		25	23	B.1.617.2	Suspected B.617.2
14966	23		15	23	B.1.617.2	Suspected B.617.2
14967	31		33	31	None	Suspected B.617.2
14968	27		29	28	None	Suspected B.617.2
14969	26		28	27	B.1.617.2	Suspected B.617.2
14970	25		28	26	B.1.617.2	Suspected B.617.2
14971	24		26	24	B.1.617.2	Suspected B.617.2
14972	32		39	32	None	Suspected B.617.2
14973	28		29	28	B.1.617.2	Suspected B.617.2
14975	23		25	24	B.1.617.2	Suspected B.617.2
14978	21		21	21	B.1.617.2	Suspected B.617.2
14980	26		28	27	B.1.617.2	Suspected B.617.2
14999	27	NA	29	29	B.1.617.2	Suspected B.617.2
15006	28	NA	30	29	B.1.617.2	Suspected B.617.2
15012	28	NA	30	29	B.1.617.2	Suspected B.617.2
15014	31	NA	32	33	None	Suspected B.617.2
15019	25	NA	28	26	B.1.617.2	Suspected B.617.2
15024	31	NA	32	32	None	Suspected B.617.2
15036	30	NA	31	31	B.1.617.2	Suspected B.617.2
15043	35	NA	36	36	None	Suspected B.617.2
15046	27	NA	28	28	B.1.617.2	Suspected B.617.2
15048	29	28	NA	NA	B.1.1	Suspected B.1.1.7
15051	28	NA	30	29	B.1.617.2	Suspected B.617.2
15053	28	NA	29	28	B.1.617.2	Suspected B.617.2
15085	33	NA	33	32	None	Suspected B.617.2

a CoV19 E, CoV19 inclusive E-sarbeco reaction; NA, no amplification.

**TABLE 3:** WGS coverage, deletions sequenced by WGS analysis, and classification of clinical samples examined using the Alpha-Delta assay

Sample^*a*^	Amino acid deletions	Coverage (%)	Pangolin clade
14918	D119-(ORF8); F120-(ORF8); F157-(S); R158-(S)	99.87	B.1.617.2
14920	E619-(S); V620-(S); P621-(S); V622-(S); A623-(S)	56.46	None
14921	D119-(ORF8); F120-(ORF8); F157-(S); R158-(S)	97.67	B.1.617.2
14922	D119-(ORF8); F120-(ORF8); F157-(S); R158-(S)	99.64	B.1.617.2
14923	D119-(ORF8); F120-(ORF8); F157-(S); R158-(S)	99.04	B.1.617.2
14924	D119-(ORF8); F120-(ORF8); F157-(S); R158-(S)	97.58	B.1.617.2
14925	D119-(ORF8); F120-(ORF8)	41.58	None
14926	D119-(ORF8); F120-(ORF8); F157-(S); R158-(S)	99.64	B.1.617.2
14928	D119-(ORF8); F120-(ORF8)	22.57	None
14929	D119-(ORF8); F120-(ORF8); F157-(S); R158-(S)	99.95	B.1.617.2
14931	D119-(ORF8); F120-(ORF8); R158-(S)		B.1.617.2
14932	D119-(ORF8); F120-(ORF8)	57.41	None
14933	D119-(ORF8); F120-(ORF8); F157-(S); R158-(S)	99.83	B.1.617.2
14934	D119-(ORF8); F120-(ORF8)		None
14936	D119-(ORF8); F120-(ORF8)	56.55	None
14937	D119-(ORF8); F120-(ORF8); F157-(S); R158-(S)	99.79	B.1.617.2
14938		57.23	None
14939	D119-(ORF8); F120-(ORF8)	68.74	None
14940	D119-(ORF8); F120-(ORF8); F157-(S); R158-(S)	97.29	B.1.617.2
14941	S3675-(ORF1a); G3676-(ORF1a); F3677-(ORF1a); H69-(S); V70-(S); Y144-(S)	99.78	B.1.1.7
14945	D119-(ORF8); F120-(ORF8)	69.39	None
14946	D119-(ORF8); F120-(ORF8); F157-(S); R158-(S)	99.18	B.1.617.2
14947	D119-(ORF8); F120-(ORF8); F157-(S); R158-(S)	98.34	B.1.404
14948	D119-(ORF8); F120-(ORF8)	60.82	None
14949	D119-(ORF8); F120-(ORF8); F157-(S); R158-(S)	99.83	B.1.617.2
14950	D119-(ORF8); F120-(ORF8); F157-(S); R158-(S)	98.94	B.1.617.2
14951	D119-(ORF8); F120-(ORF8)	36.15	None
14952	D119-(ORF8); F120-(ORF8); F157-(S); R158-(S)	98.97	B.1.617.2
14953	D119-(ORF8); F120-(ORF8); F157-(S); R158-(S)	99.82	B.1.617.2
14954	D119-(ORF8); F120-(ORF8); F157-(S); R158-(S)	99.45	B.1.617.2
14955	D119-(ORF8); F120-(ORF8); F157-(S); R158-(S)	99.72	B.1.617.2
14957		20.91	None
14958	D119-(ORF8); F120-(ORF8); F157-(S); R158-(S)	99.8	B.1.617.2
14960	D119-(ORF8); F120-(ORF8); F157-(S); R158-(S)	99	B.1.617.2
14963	D119-(ORF8); F120-(ORF8)	69.01	None
14965	D119-(ORF8); F120-(ORF8); F157-(S); R158-(S)	99.73	B.1.617.2
14966	D119-(ORF8); F120-(ORF8); F157-(S); R158-(S)	99.61	B.1.617.2
14967	D119-(ORF8); F120-(ORF8)	70.23	None
14968	D119-(ORF8); F120-(ORF8)		None
14969	D119-(ORF8); F120-(ORF8); F157-(S); R158-(S)		B.1.617.2
14970	D119-(ORF8); F120-(ORF8); F157-(S); R158-(S)	98.53	B.1.617.2
14971	D119-(ORF8); F120-(ORF8); F157-(S); R158-(S)	99.9	B.1.617.2
14972	C1732-(ORF1b); L1733-(ORF1b); C1734-(ORF1b)	36.73	None
14973	D119-(ORF8); F120-(ORF8); F157-(S); R158-(S)	97.09	B.1.617.2
14975	D119-(ORF8); F120-(ORF8); F157-(S); R158-(S)	99.55	B.1.617.2
14978	D119-(ORF8); F120-(ORF8); F157-(S); R158-(S)	99.68	B.1.617.2
14980	D119-(ORF8); F120-(ORF8); F157-(S); R158-(S)	99.82	B.1.617.2
14999	D119-(ORF8); F120-(ORF8); F157-(S); R158-(S)	98.63	B.1.617.2
**Sample**	**Amino acid deletions**	**Coverage (%)**	**Pangolin clade**
15006	D119-(ORF8); F120-(ORF8); F157-(S); R158-(S)	97.86	B.1.617.2
15012	D119-(ORF8); F120-(ORF8); F157-(S); R158-(S)	94.57	B.1.617.2
15014	D119-(ORF8); F120-(ORF8)		None
15019	L206-(M); D119-(ORF8); F120-(ORF8); F157-(S); R158-(S)		B.1.617.2
15024	D119-(ORF8); F120-(ORF8); F157-(S); R158-(S)	94.55	B.1.617.2
15036	D119-(ORF8); F120-(ORF8); F157-(S); R158-(S)	97.86	B.1.617.2
15043	D119-(ORF8); F120-(ORF8)	47.82	None
15046	D119-(ORF8); F120-(ORF8); F157-(S); R158-(S)	97.87	B.1.617.2
15048	H69-(S); V70-(S); Y144-(S)	98.28	B.1.1
15051	D119-(ORF8); F120-(ORF8); F157-(S); R158-(S)	98.38	B.1.617.2
15053	D119-(ORF8); F120-(ORF8); F157-(S); R158-(S)	91.98	B.1.617.2
15085	D119-(ORF8); F120-(ORF8)	33.42	

a Alpha samples are highlighted in gray.

### Detection of the Alpha and Delta lineage in wastewater samples.

The capability of the Alpha-Delta and confirmatory Orf8_119del_ assays to detect the presence of the Delta lineage RNA in sewage samples was evaluated using samples from different periods of the COVID-19 pandemic, from December 2020 to mid-June 2021. As detailed in Table 4, the assay correctly identified the samples from 2020 as non-Alpha and non-Delta. Samples collected during the peak of the third morbidity wave in Israel were all identified as positive for the Alpha variant, and samples collected from July 2021 were all identified as positive for the Delta variant (Table 4). The *C_q_* values of the N_D3L_ and S_157del_ reactions, when positive, were comparable to those of the E-sarbeco reaction, demonstrating sufficient sensitivity of these reactions with RNA extraction of wastewater. These results indicated that the Alpha-Delta assay is sufficiently sensitive to detect the presence of SC-2 in wastewater and determine whether the Alpha or Delta lineages or both are circulating in the examined regions. In order to evaluate the capability of the ORF8_119del_ reaction to correctly detect the Delta linage RNA in wastewater samples, it was applied to samples that were previously identified as non-Alpha, non-Delta, or as Delta positive. Samples selected randomly from 2020 were all negative for the presence of the ORF8_119del_ mutation, while recent samples from July 2021 were all identified as positive for the mutation (Table 5). Although the *C_q_* values obtained with the ORF8_119del_ reaction were 2 to 3 cycles higher than those of the S_157del_ reaction in this test, all samples were correctly identified. Collectively, these results show that both the Alpha-Delta multiplex assay and the confirmatory ORF8_119del_ test can be used for examination of identification of the Alpha and Delta lineages in wastewater samples.

**TABLE 4:** Detection of Alpha and Delta lineage in wastewater samples using the Alpha-Delta assay

Sample	CoV19 E	N_D3L_	S_157del_
September to December 2020^*a*^^,^^*b*^			
S-2299	35	NA	NA
S-2302	32	NA	NA
S-2307	32	NA	NA
S-2310	30	NA	NA
S-2314	31	NA	NA
S-2318	30	NA	NA
S-2328	31	NA	NA
S-2332	31	NA	NA
S-2336	31	NA	NA
S-2340	32	NA	NA
S-2344	30	NA	NA
S-2349	33	NA	NA
S-2353	32	NA	NA
S-2357	32	NA	NA
S-2382	30	NA	NA
S-2386	31	NA	NA
S-2390	30	NA	NA
S-2394	34	NA	NA
S-2399	31	NA	NA
S-2402	32	NA	NA
S-2407	31	NA	NA
S-2424	30	NA	NA
S-2457	32	NA	NA
S-2461	34	NA	NA
S-2480	32	NA	NA
S-2493	33	NA	NA
S-2498	30	NA	NA
S-2501	29	NA	NA
S-2505	31	NA	NA
S-2509	33	NA	NA
February to March 2021^*a*^^,^^*b*^			
S-7369	31.50	32.74	NA
S-7419	31.36	33.01	NA
S-7451	31.79	33.40	NA
S-7455	31.01	33.16	NA
S-7459	30.63	31.79	NA
S-7463	30.83	32.03	NA
S-7467	31.26	32.39	NA
S-7592	31.21	32.19	NA
S-7600	31.11	32.39	NA
S-7604	31.76	33.44	NA
S-7613	32.14	33.61	NA
S-7678	32.75	34.74	NA
S-7699	29.06	30.15	NA
S-7718	32.71	34.16	NA
S-7722	31.89	33.72	NA
S-7727	31.78	33.10	NA
S-7734	32.72	33.49	NA
S-7742	32.71	33.92	NA
S-7754	32.02	33.40	NA
S-7762	32.07	33.69	NA
S-7369	31.50	32.74	NA
S-7419	31.36	33.01	NA
S-7451	31.79	33.40	NA
S-7455	31.01	33.16	NA
S-7459	30.63	31.79	NA
S-7463	30.83	32.03	NA
S-7467	31.26	32.39	NA
S-7592	31.21	32.19	NA
July 2021^*a*^^,^^*b*^			
S-12689	28	NA	29
S-12694	28	NA	29
S-12698	30	NA	31
S-12733	28	NA	29
S-12737	26	NA	27
S-12741	28	NA	28
S-12745	29	NA	30
S-12754	27	NA	28
S-12762	30	NA	30
S-12766	29	NA	29
Env_128	31	NA	29
Env_129	33	NA	31
Env_130	31	NA	30
Env_134	33	NA	30
Env_138	32	NA	29
Env_140	30	NA	28
Env_143	31	NA	30
Env_144	29	NA	28
Env_145	30	NA	29
Env_146	36	NA	33
Env_152	35	NA	30
Env_163	29	NA	30
Env_168	32	NA	29
Env_172	33	NA	32
Env_173	30	NA	31
Env_177	30	NA	32
Env_186	30	NA	31
Env_197	29	NA	29
Env_198	30	NA	30
S-12686	31	NA	31

a Samples were collected during September to December 2020, February to March 2021, and July 2021, and the *Cq* values for each reaction are shown.

b NA, no amplification.

**TABLE 5:** Detection of Delta lineage in wastewater samples using the Orf8_119del_ test

Sample	CoV19 E	S_157del_	Orf8_119del_
September to December 2020^*a*^^,^^*b*^			
S-2480	32	NA	NA
S-2493	33	NA	NA
S-2498	30	NA	NA
S-2501	29	NA	NA
S-2505	31	NA	NA
S-2509	33	NA	NA
July to August 2021^*a*^^,^^*b*^			
12689	28	29	31
12694	28	29	31
12698	30	31	32
12733	28	29	31
12737	26	27	30
12741	28	28	31
12745	29	30	32
12754	27	28	30
12762	30	30	32
12766	29	29	32
Env_128	31	29	32
Env_129	33	31	34
Env_130	31	30	32
Env_134	33	30	34
Env_138	32	29	31
Env_140	30	28	31
Env_143	31	30	33
Env_144	29	28	30
Env_145	30	29	31
Env_146	36	33	33
Env_152	35	30	33
Env_163	29	30	32
Env_168	32	29	32
Env_172	33	32	35
Env_173	30	31	34
Env_177	30	32	34
Env_186	30	31	34
Env_197	29	29	32
Env_198	30	30	33
S-12686	31	31	34

a Randomly selected wastewater samples that were previously tested with the Alpha-Delta assay were subsequently examined using the confirmatory Orf8_119del_ test, and the *Cq* values for each reaction are shown.

b NA, no amplification.

## DISCUSSION

The emergence of SC-2 variants that have the potential to affect both COVID-19 spread and vaccine evasion poses additional challenges for current intensive diagnostic efforts worldwide. The primary goal of diagnostic SC-2 tests was initially to detect the presence of SC-2 RNA, either in clinical or environmental samples. However, the need for rapid classification of SC-2 samples for their lineage, once a sample is determined as SC-2 positive, becomes essential when attempting to monitor emergence or incursion of new variants into a country, a geographic region, or a community. Surveillance through sequencing is currently the ultimate tool to identify the emergence of new and potentially important variants (20). However, whole-genome sequencing (WGS) is expensive and time-consuming, requires exceptionally skilled personnel, and cannot be readily scaled-up for high-throughput testing compared to rapid molecular tests (16, 21). In this dynamic situation of emerging variants, rapid, cost-effective, and high-throughput identification of circulating SC-2 variants is required.

We recently reported on the development and utilization of a rapid multiplex RT-qPCR assay that successfully classified SC-2 samples as Alpha, Beta, or neither. We demonstrated that it could be readily scaled-up to accommodate high-throughput testing (17). Here, we describe a new assay that combines general detection of SC-2, together with specific classification of the two major variants Alpha and Delta. Previous reports of in-house-selective assays, as well as commercial variant detection kits, rely mostly on the identification of common mutations that are not specific to a particular variant (22–24). Other variant of concern (VOC) detection approaches rely on multistep expensive procedures (25, 26). Such assays are not suitable for rapid determination of the identity of an examined sample based on a single assay. Confirmation of the Alpha-Delta assay results by Sanger and Illumina sequencing reinforced its importance as a first-line rapid and reliable tool for classification of major VOCs, obviating the need for further examination. The identification of both Orf8 and spike gene Delta-specific deletions in samples with insufficient sequencing coverage demonstrated the usefulness of the Alpha-Delta assay in determining the identity of “sequencing-resistant” samples. Such problems can result from low RNA quantity (as evident by the *C_q_* values detailed in Table 2) or other factors that inhibit the sequencing procedure. This is particularly important in classification of wastewater-derived samples, where the target RNA is a pool from multiple individuals, is diluted, and the samples contain chemical inhibitors. The Alpha-Delta assay successfully detected the presence of the Delta variant even in very low RNA concentration, thereby enabling sensitive monitoring of the circulation of this variant during its incursion into Israel. This assay was also used for screening international arrivals, which is particularly important when attempting to prevent the spreading of new variants. While other reported approaches to detect SC-2 variants in international travelers rely on lengthy multistep procedures (25, 26), this assay is rapid (∼70 min from extraction to answer) and can be implemented in most standard laboratories that perform routine PCR tests. This is especially advantageous when an accelerated epidemiological investigation or other actions need to be commenced and complete genomic analysis is not available.

The Orf8_119del_ reaction was used in this study as a confirmatory assay, complementing the first-line Alpha-Delta assay. This is useful to confirm ambiguous results and to provide additional support for lineage classification when sequencing analysis is not available or insufficient. The S_157del_ and the Orf8_119del_ reactions showed similar performance in both clinical and wastewater samples and can therefore be used in the current situation where both deletions are unique for the Delta variant. However, if a new variant will emerge where one of these mutations is present, reassessment of the multiplex combination should be performed in order to adjust the reactions to the required assay specificity. The ability to assemble different reactions into a single multiplex assay is advantageous with respect to the intended use of the assay, as we show here. When the Beta variant was introduced in Israel, we used the combination of the Alpha Beta and inclusive E reactions to classify the examined samples. Upon introduction of the Delta variant, we modified the assay and added a confirmatory reaction to address the pressing need to identify the Delta and Alpha variants but also identify non-Alpha- and non-Delta-positive samples, all in a single 80-min standard RT-qPCR assay. The implementation of such a self-developed method is limited to laboratories that are able to combine research with diagnostic routine rather than high-throughput diagnostic laboratories. Another limitation is the rapid pace in which novel variants are emerging, which, in some cases, may obviate the need to implement such selective assays in routine diagnostic work. The inability to predict which emerging variants will become widespread poses a major obstacle when having to decide which assays to develop.

Nevertheless, this study demonstrates that in addition to implementation of a useful diagnostic tool, the approach of developing a tailor-made variant-specific qPCR assay can improve the SC-2 diagnostic capability in any qPCR-qualified laboratory. Moreover, it can complement variant classification when WGS analysis either fails or is not available. Such an assay can therefore be valuable for epidemiological studies, enabling a quick and affordable assessment of the circulation of major variants in a community or in a geographic region of interest. It is conceivable that the emergence of future SC-2 variants, and possibly other infectious viruses, will warrant further development of such diagnostic tools.

## MATERIALS AND METHODS

### Ethics statement.

The study was conducted according to the guidelines of the Declaration of Helsinki and was approved by the Institutional Review Board of the Sheba Medical Center (7045-20-SMC). Patient consent was waived as the study used remains of clinical samples, and the analysis used anonymous clinical data.

### Preparation of clinical and environmental samples.

Clinical samples were prepared during the laboratory diagnostic routine from nasopharyngeal swabs, as described before (17). RNA was extracted using either the MagNA Pure 96 system (Roche) or the PPS MagLEAD system. The extractions were used for routine clinical diagnostics and were incubated at −80°C for long-term storage prior to the novel assay development. Environmental samples were processed as described before (6, 19). Briefly, sewage samples were centrifuged and filtered before extraction with either Nuclisense EasyMag (bioMérieux) or EMag extraction systems. Immediately after extraction, all samples were preserved at −80°C until PCR testing was performed.

### Cell culture.

All SC-2 culturing experiments were performed under biosafety level 3 (BSL3) conditions according to the Sheba Medical Center biosafety guidelines. In order to culture positive SC-2 samples, the medium from the selected collection tubes was filtered using a 0.22-μm filter (Millipore) and used for inoculation of Vero E6 cells. The cells were allowed to reach 70% confluency and were then incubated for 1 h at 37°C with 300 μL of the filtered medium. After 1 h, the medium was removed, and the cells were incubated in modified Eagle medium (MEM-EAGLE) with 2% fetal calf serum (FCS). Cells were monitored daily for the presence of cytopathic effect (CPE). Upon CPE onset, the culture medium was collected, and viral RNA was extracted. Resulting RNA extractions were kept at −80°C until use.

### Design of the B.1.671-specific RT-qPCR assays.

Sequences classified as B.1.617 were obtained from the GISAID database (https://www.epicov.org/epi3/frontend#2d1c1e) and were analyzed by alignment with reference sequence NC_045512 to identify mutated regions that can be used for differential analysis. Uniqueness of the selected mutations was confirmed by performing global analysis for the selected mutations in the NextStrain website to identify other lineages with identical mutations (https://nextstrain.org/ncov/gisaid/global). Corresponding primers and probes that detect only the mutated sequences were designed for each region and examined *in silico* for secondary structure formation, specificity, and compatibility with qPCR assays using Geneious software and the NCBI BLAST (https://blast.ncbi.nlm.nih.gov/Blast.cgi). The primers and probes used in this study are detailed in Table 1.

### RT-qPCR assay.

MDX106 inhibitor-tolerant RT-qPCR mix was from Bioline. Primers and probes were either from Metabion or from Merk-Sigma Israel. Reaction components were assembled as described in Table 6 using the MDX106 inhibitor-tolerant mix. The reactions were run in a CFX-96 thermal cycler (Bio-Rad) using the following parameters: 45°C for 10 min 10 s, 45 cycles of 95°C for 2 min 20 s, 95°C for 4 s, and 60°C for 22 s. Fluorescence was recorded at 60°C at the end of each amplification cycle. Results were analyzed using the CFX Maestro software (Bio-Rad).

**TABLE 6:** Reaction mix composition for the S_157del_ and Orf8_119del_ assays

S_157del_ assay		
E + S_157del_ + N_D3L_+ RNase P multiplex mix	Final concn	Vol/reaction (μL)
Meridian MDX 4× mix	1×	5
H_2_O		3.73
22003 Fwd 40 μM	500 nM	0.3
22075 Rev 40 μM	500 nM	0.3
21987 probe 20 μM	250 nM	0.3
E-Sarbeco-F1b 40 μM	400 nM	0.25
E-Sarbeco-R 40 μM	400 nM	0.25
E-Sarbeco-P FAM 20 μM	200 nM	0.3
28257 N VOC Fwd 40 μM	600 nM	0.3
2019-nCoV_N1-R 40 μM	600 nM	0.3
2019-nCoV_N1-P HEX 20 μM	300 nM	0.3
RNasP-F	300 nM	0.25
RNasP-R	300 nM	0.25
RNasP-P/Cy5	300 nM	0.17
Total master mix vol		12
RNA sample		8
Total reaction vol		20
**Orf8_119del_ assay**		
**E + Orf8_119del_ multiplex mix**	**Final concn**	**Vol/reaction (μL)**
Meridian MDX 4× mix	1×	5
H_2_O		5.65
28225 Fwd	500 nM	0.25
28232 Rev 40 μM	500 nM	0.25
28199 Probe HEX 20 μM	250 nM	0.25
E-Sarbeco-F1b 40 μM	400 nM	0.2
E-Sarbeco-R 40 μM	400 nM	0.2
E-Sarbeco-P FAM 20 μM	200 nM	0.2
Total master mix vol		12
RNA sample		8
Total reaction vol		20

### Sanger sequencing of culture-derived and clinical samples.

The N-terminal domain (NTD) of the SC-2 spike gene and the C-terminal domain (CTD) of SC-2 Orf8 were amplified using the primer pairs detailed in Table 1. Primer pair T7-21494 Fwd + Cov19 22475 was used for the spike region amplification, and primer pair pT7 27945 Fwd + 28525 Rev was used for the Orf8 region amplification. PCR was performed using the PCRBIO 1-step go reverse transcription-PCR (RT-PCR) kit according to the manufacturer’s instructions. The PCR products were resolved by 1.2% agarose gel electrophoresis to confirm adequate amplification. Each product was then used as the template for the dye labeling reaction using the BigDye kit according to the manufacturer’s instructions. Sequencing was performed using the ABI 3500 genetic analyzer.

### Generation of *in vitro* transcribed standard RNA.

RNA standards of the reaction targets were designed and synthesized as described before (17). The primers for the amplification of the S_157del_ and Orf8_119del_ reaction targets are detailed in Table 1. RT-PCR was performed with Fwd primers containing the minimal T7 promoter sequence. Resulting PCR products were purified using the Macherey-Nagel NucleoSpin gel and PCR clean-up kit according to the manufacturer’s instructions. The purified products were then used for *in vitro* transcription with the T7-Megascript kit according to the manufacturer’s instructions (Thermo Fisher). The concentration of the resulting RNA was measured using a NanoDrop spectrophotometer. All reaction products were kept at −80°C until use.

### Evaluation of analytical and clinical sensitivity of the Alpha-Delta assay.

In order to facilitate rapid classification of the examined sample, four reactions were combined in a single assay, as detailed below. The inclusive E-sarbeco, which detects SC-2 RNA regardless of the variant (6-carboxyfluorescein [FAM] fluorophore), N_D3L_ reaction, which detects the Alpha variant (HEX channel), S_157del_ reaction, which detects the Delta variant (Texas Red channel), and an endogenous control reaction for the human RNase P gene (Cy5 channel). The details of the E, N_D3L_, and RNase P reactions were described previously (17). The combined multiplex was termed Alpha-Delta assay. The analytical sensitivity of the Alpha-Delta assay was evaluated using serial dilutions of the *in vitro* transcribed targets (IVT) of each reaction. All four targets were mixed together in an initial concentration of 10^−4 ^ng/μL, and 10-fold dilutions were prepared in nuclease-free Tris-EDTA (TE) buffer, pH 7.5 (IDT). Validation of the IVT standards was performed by running the E-sarbeco reaction with IVT dilutions together with dilutions of a commercial SC-2 RNA standard (Vircell). The analytic sensitivity of wastewater samples was evaluated by spiking IVT pool dilutions into SC-2-negative wastewater extraction, as described previously (19).

The clinical sensitivity was evaluated by serially diluting positive samples in an extraction of a SC-2-negative sample. Conversion of each *in vitro* transcribed RNA product from nanograms to copies was performed using the SciencePrimer website calculator (http://www.scienceprimer.com/) according to the RNA molecule size (Fig. S1 in the supplemental material). For target concentration of 500 copies or higher per reaction, 10 repeats were tested. For less than 500 copies per reaction, at least 20 repeats were tested for each target.

### Collection and preparation of wastewater samples.

Wastewater collection was based on composite automated sampling for 24 h located in different wastewater treatment plants. The samples were transferred under cold conditions to the lab and were processed within 24 h. Prior to further processing, culture-derived, inactivated Coronavirus OC43 strain (COV OC43) was added to the medium as described previously (6, 19). This was then used as an internal control for the concentration and extraction stages using the OC43 qPCR assay described previously (27). The concentration process uses 20 mL of raw sewage in duplicates. The raw sewage was centrifuged at 4,700 × *g* for 5 min. The supernatant was transferred to a new tube containing 0.5 g MgCl_2_ (0.26 M). The tube was gently shaken for 5 min and then filtered through 0.45-μm pore-size, 47-mm diameter electronegative mixed cellulose ester membranes (MCE) membranes (Merck Millipore Ltd.). The membrane was immediately transferred to a new tube containing 3 mL of lysis buffer (NucliSENS easyMAG). The tube was gently shaken to extract the trapped RNA virus. Total nucleic acids were extracted using the NucliSENS easyMAG system (bioMérieux, Marcyl'Etoile, France) according to the manufacturer’s instructions. Extracted nucleic acids were eluted in 55 μL of elution buffer and stored at −70°C until sequencing. All samples were tested for the presence of COV OC43 to verify proper concentration and extraction and absence of significant PCR inhibitors.

### Whole-genome sequencing.

A COVID-seq kit was used for library preparation as per the manufacturer’s instructions (Illumina). Library validation and mean fragment size were determined by Tapestation 4200 via a DNA HS D1000 kit (Agilent). Libraries were pooled, denatured, and diluted to 10 pM and sequenced on a NovaSeq system (Illumina).

### Data availability.

The nucleotide sequence data of the SARS-CoV-2 variants were deposited in GenBank under accession numbers OM766203 to OM766210.
