# Supplementary material for: Specific Detection of SARS-CoV-2 Variants B.1.1.7 (Alpha) and B.1.617.2 (Delta) Using a One-Step Quantitative PCR Assay
Source: Microbiol Spectr. 2022 Mar 14;10(2):e02176-21. doi: 10.1128/spectrum.02176-21 (PMC9045307; doi:10.1128/spectrum.02176-21)
Supplement: SUPPLEMENTAL FILE 1 — Supplemental material. Download SPECTRUM02176-21_Supp_1_seq1.pdf, PDF file, 1.0 MB [file spectrum02176-21_supp_1_seq1.pdf]

SUPPLEMENTARY MATERIAL

SPECIFIC DETECTION OF SARS-COV-2 VARIANTS B.1.1.7 (ALPHA) AND B.1.617.2 (DELTA) USING A ONE-STEP QUANTITATIVE PCR ASSAY

Oran Erster[1§], Ella Mendelson[1,2], Areej Kabat[1], Virginia Levy[1], Batya Menasheh[1], Hadar Assraf[1], Roberto Azar[1], Yaniv Ali[1], Efrat Bucris[1], Dana Bar-Ilan[1], Orna Mor[1,2], Michal Elul[1], Michal Mandelboim[1,2], Danit Sofer[1], Shai Fleishon[1], Neta S Zuckerman[1‡], Itay Bar-Or[1‡]

[1]Central Virology Laboratory, Public Health Services, Ministry of Health, Chaim Sheba Medical Center, Ramat Gan, Israel.
[2]School of Public Health, Sackler Faculty of Medicine, Tel-Aviv University, Tel-Aviv, Israel.
[§]Corresponding author contact information: oran.erster@sheba.health.gov.il
[‡]These authors contributed equally to this study

Target	Molecule size (bp)	Copies in 0.08 ng
COV19 E	422	3.4E+08
N_{D3L}	1100	1.3E+08
S_{157del}	966	1.5E+08
$Orf8_{119del}$	600	2.4E+08
RNAse P	400	3.5E+08

Supplementary Table S1. Conversion of the *in vitro*-transcribed calibration RNA target values. The mass of each target RNA molecule was converted to copies with the SciencePrimer calculator (http://www.scienceprimer.com/), using the ssRNA conversion algorithm.

NCOV-E reaction

Copies/RXN	average	stdev
3.33E+06	18.64	0.07
3.33E+05	22.13	0.08
3.33E+04	26.06	0.16
1.20E+04	26.41	0.11
5.00E+03	29.0	0.22
1.20E+03	29.61	0.03
5.00E+02	32.3	0.24
1.20E+02	33.45	0.50
5.00E+01	35.9	1.59
1.20E+01	36.20	0.76
5.00E+00		

Orf8$_{19del}$ reaction

Copies/RXN	average	Stdev
2.36E+06	18.96	0.54
2.36E+05	22.25	0.21
5.00E+04	23.95	0.27
2.36E+04	26.17	0.53
5.00E+03	27.11	0.08
5.00E+02	29.97	0.23
5.00E+01	32.96	1.09
2.36E+01	35.84	0.63
5.00E+00		

N$_{D3L}$ reaction

Copies/RXN	average	Stdev
1.29E+07	15.69	0.250
1.29E+06	19.31	0.039
1.29E+05	23.49	0.043
1.29E+04	26.37	0.104
5.00E+03	28.2	0.244
5.00E+02	31.5	0.367
5.00E+01	35.5	1.170
5.00E+00		

hRNASE P reaction

Copies/RXN	average	Stdev
3.54E+07		
3.54E+06	18.68	0.17
3.54E+05	22.10	0.17
3.54E+04	26.30	0.18
5.00E+03	29.4	0.18
5.00E+02	32.6	0.46
5.00E+01	35.9	0.76
3.54E+00		

S$_{157del}$ reaction

Copies/RXN	average	Stdev
1.45E+06	18.24	0.05
1.45E+05	21.48	0.11
1.45E+04	25.57	0.11
5.00E+03	28.18	0.25
5.00E+02	31.06	0.23
5.00E+01	33.91	0.95
1.45E+00	39.37	1

Supplementary Table S2. Average Cq and standard deviation (Stdev) values of the Alpha-Delta reactions calibration curves. The average value for each dilution was calculated for each reaction. For [copies/reaction] down to $5X10^3$, ten replicates were tested. For dilutions between $5X10^2$ and $5X10^0$ [copies/reaction], twenty replicates were tested for each dilution. The cells with grey background contain data obtained with a commercial SC-2 RNA standard (https://en.vircell.com/products/).

**Sample**	**COV19 E**	**S_{157del}**	**$Orf8_{119del}$**
WT 1	27.36	N/A	N/A
WT 2	31.1	N/A	N/A
WT 3	25.27	N/A	N/A
WT 4	24.99	N/A	N/A
WT 5	28.04	N/A	N/A
B.1.1.7 - 1	22.08	N/A	N/A
B.1.1.7 - 2	18.73	N/A	N/A
B.1.1.7 - 3	24.61	N/A	N/A
B.1.351 - 1	19.3	N/A	N/A
B.1.351 - 2	28.28	N/A	N/A
B.1.351 - 3	20.34	N/A	N/A
B.1.351 - 4	29.9	N/A	N/A
P1	32.15	N/A	N/A

Supplementary Table S3. Specificity evaluation of the B.1.617.2 S_{157del} and the $Orf8_{119del}$ reactions. Samples from indicated lineages were examined using a duplex assay consisting of the inclusive E-sarbeco reaction, and the specific $S_{156\text{-}157del}$ reaction and a different duplex assay containing the E-sarbeco and the $Orf8_{119del}$ reactions. The Cq values obtained for the E-sarbeco reaction are from the E+S_{157de}l test. N/A – No Amplification.

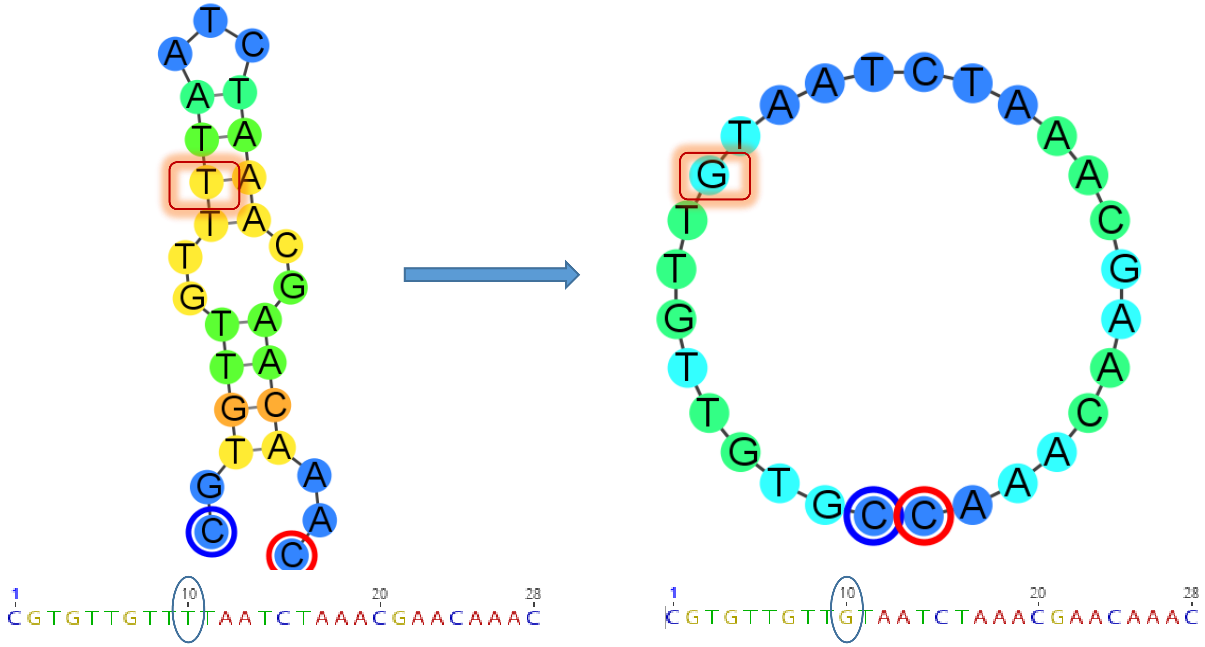

<u>Supplementary Figure S1.</u> Design of the Orf8119-120del probe. In order to avoid secondary structure formation of the 28199 probe, the deoxythymidine base was replaced with deoxyguanine, thereby destabilizing the secondary structure and enabling annealing of the probe to the target sequence. The base replacement positions are circled. The simulation temperature was 60°C. The simulation was performed using the Geneious software (www.geneious.com).

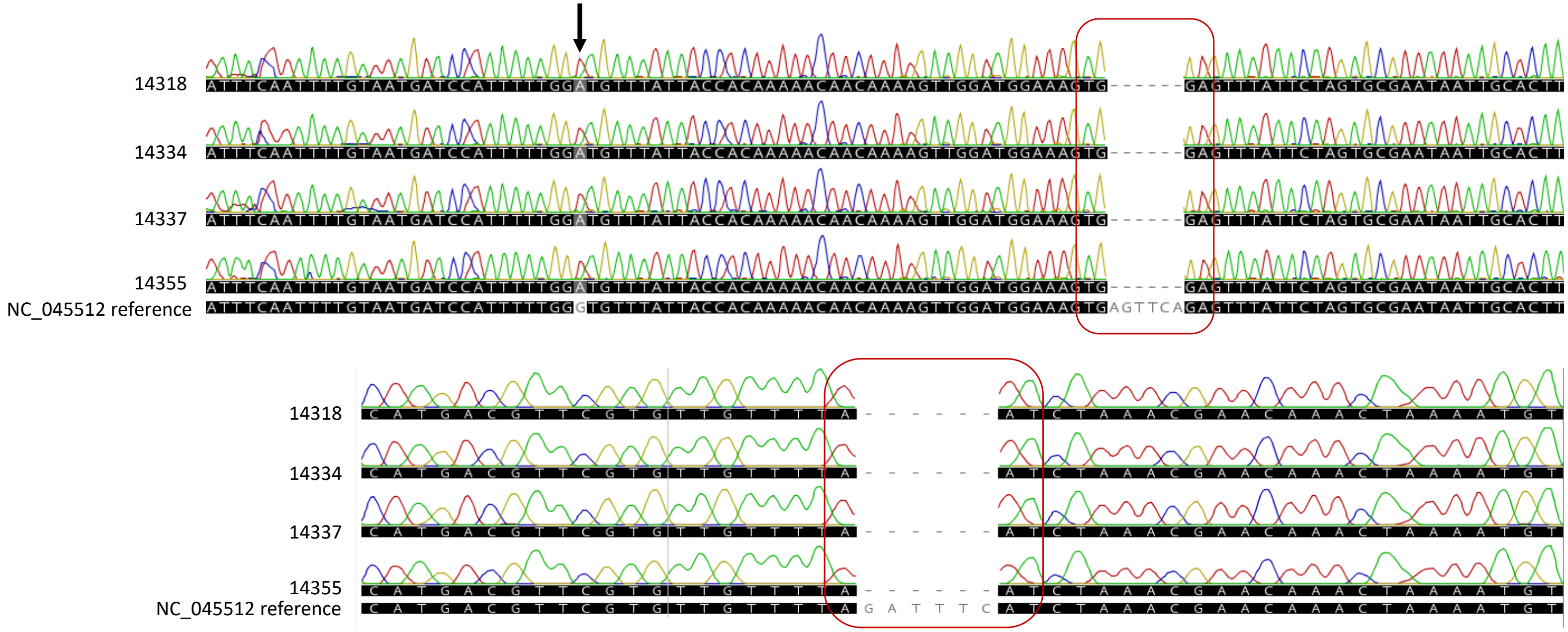

<u>Supplementary Figure S2.</u> Sanger Sequencing of B.1.617-suspected samples. The B.1.617-specific deletion regions of four samples identified as “B.1.617-suspected” by the S_{157del} and $Orf8_{119del}$ reactions were sequenced. (A) Alignment of the S_{157del} region with reference sequence NC_045512. The deletion region is marked with a rectangle. The A to G substitution, which translates into G142D amino acid mutation, is marked with an arrow. (B) Alignment of the $Orf8_{119del}$ region with reference sequence NC_045512. The deletion region is marked with a rectangle.

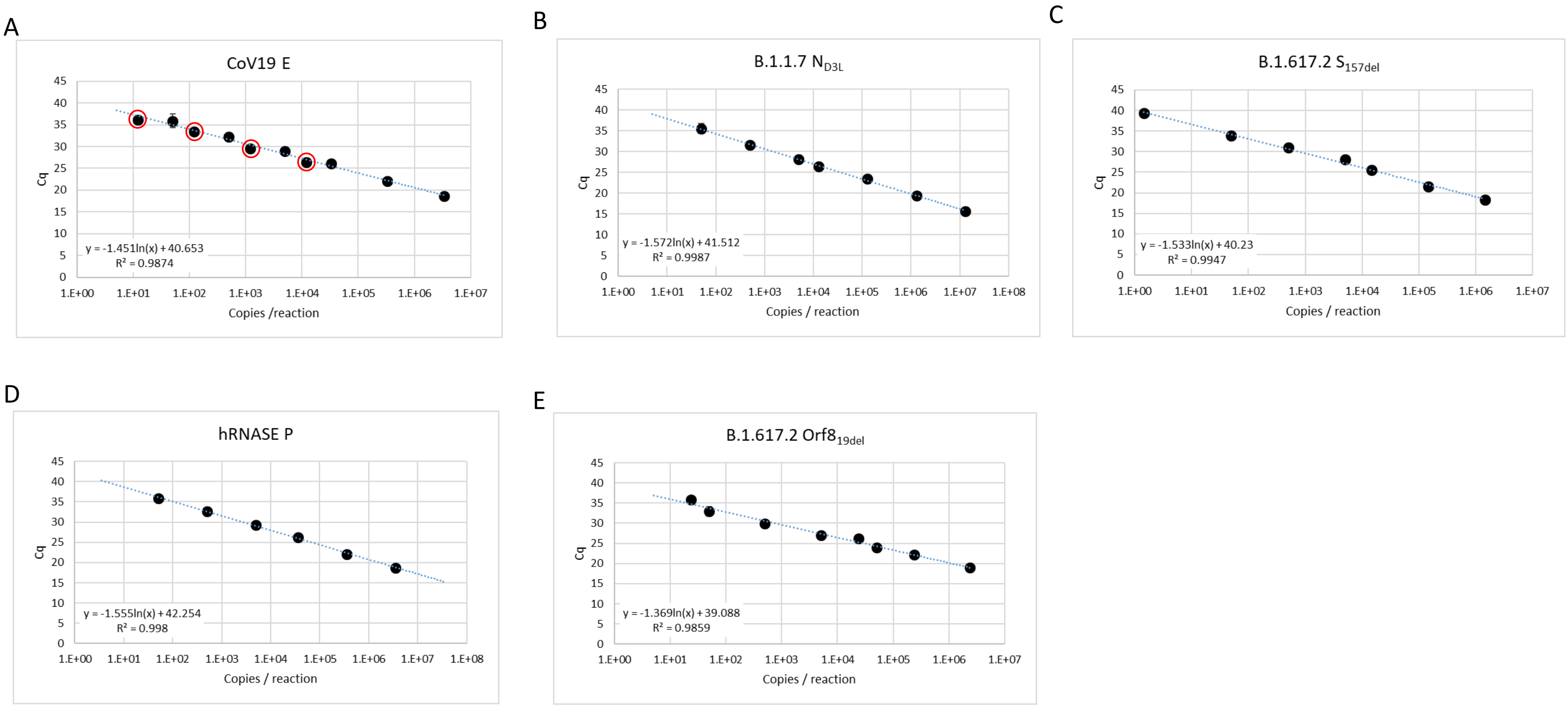

Supplementary Figure S3. Analytical Limit of Detection (LOD) of the Alpha-Delta assay. In vitro transcribed RNA targets of the four assay reactions were pooled and then diluted 10-fold. The average Cq value of each target for each dilution was plotted against the calculated target concentration. (A) E-sarbeco reaction combined plot. The plot combines values obtained from the In vitro transcribed pool dilutions and commercial SC-2 RNA dilutions (Circled in red). (B) ND3L reaction plot. (C) S157del plot. (D) hRNAse P plot. The 5X103 copies/reaction dilution of each reaction was run in 16 replicates and the last three dilutions (5X102 – 5X100 copies/reaction) were run in twenty replicates. The derived regression curve, its formula and R2 value are shown for each graph.

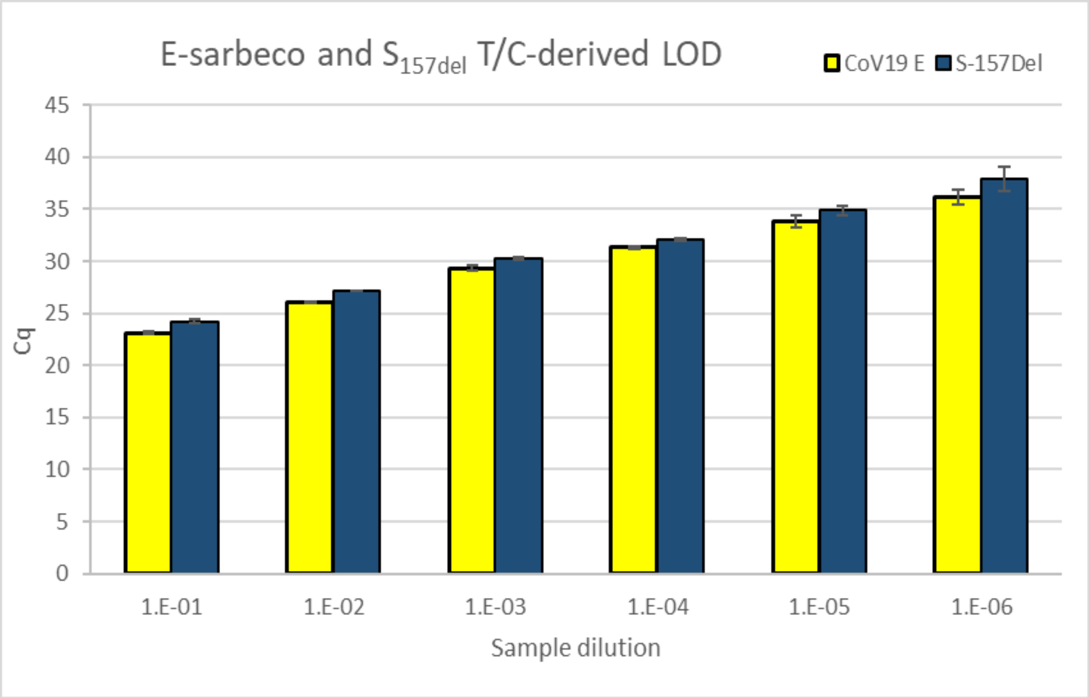

Supplementary Figure S4. Evaluation of the the S_{157del} reaction sensitivity with culture-derived virus. RNA was extracted from cultured B.1.617.2 strain and serial dilutions were prepared as indicated. The dilutions were tested using the Alpha-Delta assay. The average Cq value and standard deviation of 3 repeats for each dilution are shown, for the inclusive E reaction and the specific S_{157del} reaction.
